# The Influence of Chronic Illness and Lifestyle Behaviors on Quality of Life among Older Thais

**DOI:** 10.1155/2016/2525941

**Published:** 2016-02-28

**Authors:** Ratana Somrongthong, Donnapa Hongthong, Sunanta Wongchalee, Nualnong Wongtongkam

**Affiliations:** ^1^College of Public Health Sciences, Chulalongkorn University, Bangkok 10330, Thailand; ^2^Boromarajonani College of Nursing, Phayao 56000, Thailand; ^3^School of Biomedical Sciences, Charles Sturt University, Bathurst, NSW 2795, Australia

## Abstract

Chronic conditions and lifestyle behaviors have a detrimental influence on the quality of life for seniors because of physical disability and emotional concerns. This study aimed to assess the influence of chronic illness, smoking, and alcohol use on quality of life among Thai seniors. A cross-sectional study was conducted in three communities, selected purposively from the North, Northeast, and Central regions, and 1278 senior participants were recruited. Binary logistic regression was used to predict the influence of factors on quality of life with adjusted covariates. Most participants were aged 60–70 years and married, earned 500–1,000 Baht/month (US $17–$35), had one chronic illness, and were nonsmokers and nondrinkers. Surprisingly, there appeared to be no link between chronic conditions and quality of life. Current drinkers were more likely to have a high quality of life, with Odds Ratios of 2.16 for men and 2.73 for women. Seniors of both genders who were current drinkers were more likely to accept death and dying and this improved their quality of life. Social participation in alcohol consumption may encourage seniors to share their concerns about death and dying and eventually accept this as a foundation of life.

## 1. Introduction

Elderly people experience a variety of chronic diseases because of biological degeneration, with health problems being almost inevitable in the last period of human life. The most frequent degenerative diseases leading to reduced quality of life (QoL) are cancer, hypertension, osteoporosis, and diabetes mellitus [1, 2]. Cancer is a leading cause of death in Thailand, with age-adjusted mortality rates of 89.7 per 100,000 in males and 67.2 per 100,000 in females. Liver cancer is the most prevalence cancer in men and the third most common cause of cancer in females [3]. Cancer survivors are affected physically, psychologically, socially, and spiritually [4], similar to the lower levels of quality of life seen in breast cancer survivors, which is a major cause of death among women [5].

The quality of life in chronic illnesses can vary with age, especially for senior adults. Chronic conditions affect seniors' mobility and consequently their physical and functional status [6, 7], emotional balance, and self-esteem decline because of their dependence on others. These, in turn, contribute to the reduction in the quality of life for seniors [8]. Review articles have shown consistently negative relationship between multiple chronic diseases and quality of life [9]. The presence of chronic illness was related to unhappiness and psychological distress, resulting in low quality of life for both men and women [10]. Nevertheless, there is inconsistency in research findings. Öztürk and colleagues found no association between chronic diseases and physical mobility, functional independent activities, and type of chronic conditions in either gender [11]. Despite these inconsistent findings, degenerative diseases still have a significant influence on quality of life.

Behavioral risk factors, such as smoking and alcohol abuse, are another important influence on quality of life. Empirical studies clearly show that alcohol and smoking habits have negative consequences for health-related quality of life by reducing life expectancy and creating psychological problems [12–14]. A cross-sectional study conducted in five regions in China found that lifestyle factors, particularly smoking and alcohol drinking, had a strong association with health-related quality of life [15]. The majority of studies related to chronic diseases and lifestyle behaviors have been conducted in developed countries. Less is known about the influence of lifestyle factors, including smoking and alcohol drinking, and chronic illnesses of aging on the quality of life in developing nations.

Thailand, a low-middle income country in Southeast Asia, faces a growing burden of chronic diseases in its older population. Chronic diseases have become a major burden on the government's budget, accounting for 60 million Baht (approximately US $2 million) in 2004 [16]. The major proportion (80%) of the health budget is allocated to inpatient care for complex chronic diseases [16]. Despite this, there have been few, if any, studies in Thailand on the effects of chronic diseases and lifestyle behaviors (smoking and alcohol abuse) on quality of life. This study aimed to explore the effects of chronic disease, smoking, and alcohol drinking on quality of life among Thai seniors in three regions, North region, Northeast region, and Central region. The outcomes may help to identify factors to enhance quality of life and understand gender-specific influences on quality of life among Thai seniors.

## 2. Methods

### 2.1. Study Participants

The community-based cross-sectional study was conducted in three communities from the North, Northeast, and Central regions. One subdistrict was chosen to represent each region, based on the population density and the mix of rural and urban characteristics. The subdistricts were “Ban Tom” in Payao province (North), “Ban Non” in Khon Kaen province (Northeast), and “Ban Moh” in Saraburi province (Central). The proportion of seniors in these areas was approximately 15% [17]. A door-to-door survey was undertaken within each subdistrict, and one person aged 60 years or above who resided in each house at the time of the survey was invited to participate in the study. A total of 1,278 seniors were recruited, with 400 from North region, 428 from Northeast region, and 450 from Central region. The study was explained to participants who gave informed consent before participation. The Ethical Review Committee for Research Involving Human Research Subjects, Health Science Group, Chulalongkorn University, Thailand, approved the study protocol.

## 3. Instruments

Four self-report questionnaires were administered in a face-to-face interview. Research assistants helped participants who were unable to read or write to complete the questionnaires.

### 3.1. Sociodemographic Characteristics

Sociodemographic variables related to region, gender, age, marital status, education levels, and incomes were measured. Income was measured in five categories, from US $15–35 to more than US $1,665 per month, and education level was classified as follows: being uneducated, primary school, secondary school, certificate level, and undergraduate/postgraduate degree.

### 3.2. Self-Rated Health

Participants were asked whether they had underlying diseases diagnosed by a medical doctor, with responses “Yes,” “No,” or “Don't know.” The level of chronicity was identified by asking “How many chronic diseases do you have?” with responses categorized as one, two, three, and more than three diseases. A nurse assisted participants with these responses, to minimize misinterpretation or misunderstanding.

### 3.3. Smoking and Drinking Habits

Smoking and drinking data measured current status, years since quitting, frequency of use, and daily consumption. Time since quitting was categorized as 1–10 years, 11–20 years, 21–30 years, and more than 30 years. The quantity of alcohol consumption was calculated based on a standard alcoholic drink, being 100–125 mL for one glass, or 750 mL for one bottle.

### 3.4. WHOQOL-Old

The instrument was developed by WHOQOL Group to measure the quality of life of older adults and contained 24 items in six domains [18]. These were sensory abilities (SA), Autonomy (AUT), Past, Present, and Future Abilities (PPF), social participation (SOP), death and dying (DAD), and intimacy (INT). Each item was rated on 5-point Likert scale ranging from 1 (not at all) to 5 (an extreme amount). SA assessed sensory abilities in taste, smell, sight, hearing, and touch that affected daily life activities. AUT indicated freedom to make decisions for individual future and controlling one's own life. PPF measured satisfaction with life at present, ability to achieve goals, and recognition received. SOP asked seniors about their ability to engage in social participation and perform activities and their level of satisfaction with their participation in social communities. Perceptions of death and dying were evaluated by the DAD aspect, while the INT domain determined quality of loving or being loved and companionship. The internal consistency of the instrument was 0.88 (Cronbach's alpha).

## 4. Data Analysis

Descriptive statistics were used to analyze sociodemographic characteristics, self-rated health, smoking, and drinking habits, which were expressed as numbers and percentages. Binary logistic regression was used to determine factors that influenced quality of life between genders, with Odds Ratios (ORs) and 95% confidence intervals. The sociodemographic indicators, province, age, marital status, income, and education level, were adjusted as covariates in the logistic regression model. Quality of life scores in all items were summed and divided into binary categories by quartile ranges, normal quality of life (lower quartile), and high quality of life (upper quartile), respectively. Drinking status was analyzed against six domains of quality of life with reference to a significant relation to quality of life as a whole.

## 5. Results

Sociodemographic characteristics are shown in Table 1. Approximately 35% of participants resided in each region, and there were nearly twice as many females as males. Most were 60–70 years old and married, with primary education, and earned incomes less than US $35 a month. Nearly 70% reported chronic illness, and of these, 55% had one chronic condition, such as heart disease, hypertension, diabetes, hyperlipidemia, or arthritis, with the others having more than one such condition. Over 70% of participants had never smoked and 15% were current smokers. Most participants who were ex-smokers had quit smoking within 1–10 years. The frequency of smoking was 1–5 times a day, with most smoking half a pack (1–10 cigarettes) a day. Almost 80% of participants were nondrinkers, 11% were ex-drinkers, and 11% were current drinkers. Most former drinkers had stopped drinking within 1–10 years. Just over half of drinkers consumed approximately one glass of alcohol a day, with others drinking more.

Table 2 shows factors affecting quality of life for men and women. Women who reported having chronic conditions were 59% less likely to have high quality of life than women who did not know their illness status (adjusted Odds Ratio = 0.41; 95% CI = 0.20–0.86). The number of chronic conditions did not have a statistically significant effect on quality of life between genders. Surprisingly, females who were current smokers were more likely to show high quality of life than nonsmokers (adjusted OR = 2.22; 95% CI = 1.13–4.35). Similarly, for both genders current drinkers were nearly three times more likely to report high quality of life compared with nondrinkers (adjusted OR = 2.16 for males; adjusted OR = 2.73 for females). Quitting smoking or drinking did not have a significant effect on quality of life.

In terms of the effect of drinking status on quality of life across the six domains, current drinkers of both sexes were more likely to have a positive perception of death and dying (DAD aspect) (adjusted OR = 1.85 for males; adjusted OR = 3.78 for females). Female current drinkers were also more likely to have higher sensory abilities than nondrinkers (adjusted OR = 2.94; 95% CI = 1.37–6.28). Participants who were ex-drinkers were 92% less likely to engage in intimacy (adjusted OR = 0.08; 95% CI = 0.01–0.64). See Table 3.

## 6. Discussion

The chronic illnesses seen in the study reflect the findings of the Indonesian national health survey, which found that hypertension, hypercholesterolemia, cardiac diseases, and arthritis were the most common illnesses, found in nearly 50% of participants (mean ± SE: 1.27 + 0.01) [19]. Our study showed similar results, with 55% of participants having at least one chronic condition. These chronic conditions are a global issue that affect people's lives in both developed and developing countries. Nevertheless, the study findings showed that the number of chronic conditions seemed not to affect quality of life. The reason may be because quality of life is not dependent solely on chronic illness, and other factors may be involved, such as illness acceptance [20, 21]. Kurpas and colleagues pointed out that illness acceptance is strongly related to age and quality of life when considering chronic conditions [22]. The high level of acceptance, in turn, enhances self-reliance and self-esteem and creates the ability to cope with chronic disease and its treatments [22]. Because of their Buddhist beliefs, Thai people have shown high resilience when faced with difficulty or distressing circumstances. Nearly 95% of Thais are Buddhist and they are taught not to be afraid of aging and dying because the circle of life includes birth, illness, and death. The law of “Karma” is that death is a normal process of life and is not the end of life but rather the beginning of a new life [23]. The acceptance of chronic illness as part of the circle of life may provide calmness and serenity, which are the foundations for happiness and high quality of life [23].

Surprisingly, both male and female seniors who were current drinkers had a strong likelihood of a high quality of life, particularly in the death and dying domain. Previous studies have consistently found that consumption of small amounts of alcohol provided beneficial effects, improving cognitive function [24], dementia [25], and longevity [26, 27]. Recently, a US study has pointed out that older men who drank alcohol showed a significant association with global health measured by SF-36, with improved mental and physical health functions, while women who drank alcohol reported significant improvement in life satisfaction and decreased depression [28]. One possible explanation for these benefits of alcohol on quality of life in the study may be reduced stress levels and enhanced engagement in social activities and social networks. Social gatherings encourage people to talk and share their concerns with the group, so senior peers might discuss death and dying in relation to getting older or nearing the end of life, which may be viewed as an acceptance of death. It is also likely that because death is defined as a natural phenomenon and the beginning of a new life in Buddhism, responding to death through calmness and a positive attitude may help them to reframe their future in a positive way. Buddhist teachings encourage seniors to prepare for letting go, even of their own lives, when death is coming, so older people may accept impermanence as a basic principle of life, and that in turn contributes to improved quality of life. Further study is needed on the reasons why seniors have more tolerance of chronic illnesses and acceptance of death than other age groups and how the Buddhist religion with its mortality awareness and acceptance affects this tolerance.

## 7. Limitations of the Study

The study was a cross-sectional study and hence it was difficult to identify causal relationships between potential factors and quality of life. The study was carried out over a short period of time and potential participants may have been missed. Furthermore, the self-report questionnaires may have been affected by recall bias especially in older participants.

## Figures and Tables

**Table 1:** Sociodemographic data.

	No.	%
Region		
Northeast	428	33.49
North	400	31.30
Central	450	35.21
Gender		
Male	485	37.95
Female	793	62.05
Ages (years)		
60–70	687	53.76
71–80	423	33.10
81–90	154	12.05
>90	14	1.10
Marital status		
Single	45	3.69
Married	600	49.26
Divorced	26	2.13
Widowed (spouse died)	479	39.33
Couple (not married)	68	5.58
Education level		
No education	253	19.80
Primary	986	77.15
Secondary	28	2.19
Certificate	8	0.63
Undergraduate or postgraduate	3	0.23
Incomes (Baht/month)		
500–1,000 ($17–$35)	707	55.32
1,000–5,000 ($35–$165)	447	34.98
5,000–10,000 ($165–$335)	93	7.28
10,000–50,000 ($335–$1,665)	29	2.27
>50,000 (>$1665)	2	0.16
Illness		
No	302	23.63
Yes	876	68.54
Don't know	100	7.82
Having chronic diseases	(*n* = 876)	
One	480	54.79
Two	259	29.57
Three	100	11.42
More than three	37	4.22
Smoking status		
Nonsmoker	927	72.54
Ever smoker (now quit)	159	12.44
Current smoker	192	15.02
Quit smoking (years)	(*n* = 159)	
1–10	94	59.12
11–20	35	22.01
21–30	20	12.58
>30	10	6.29
Smoking frequency	(*n* = 192)	
1–5 times/day	119	74.38
5–10 times/day	33	20.63
>10 times/day	8	5
*Missing data*	*32*	*16.67*
Quantity of cigarettes (a day)	(*n* = 192)	
1–10	170	90.43
11–20	16	8.51
>20	2	1.06
*Missing data*	*4*	*2.08*
Drinking status		
Nondrinker	986	77.15
Ever drinker (now quit)	144	11.27
Current drinker	148	11.58
Quit drinking (years)	(*n* = 144)	
1–10	95	66.43
11–20	28	19.58
21–30	15	10.49
>30	5	3.50
*Missing data*	*1*	*0.69*
Drinking frequency	(*n* = 148)	
Once a month	46	31.29
1–3 times a week	48	32.65
3–5 times a week	26	17.69
5–7 times a week (almost every day)	27	18.37
*Missing data*	*1*	*0.68*
Quantity of alcohol consumption	(*n* = 148)	
100–125 ml (one glass)	65	56.52
150–190 ml (1/4 a bottle)	29	25.22
190–375 ml (1/2 a bottle)	13	11.30
375–565 ml (3/4 a bottle)	2	1.74
750 ml or more (1 bottle or more)	6	5.22
*Missing data*	*33*	*22.30*

**Table 2:** Comparison of quality of life between genders by binary logistic regression.

	Quality of life (QoL)			
	Male		Female	
	Crude OR	Adjusted OR	Crude OR	Adjusted OR
Illness				
Don't know	*Reference*			
No	1.19 (0.55–2.55)	1.13 (0.42–3.00)	1.15 (0.58–2.27)	0.84 (0.35–2.00)
Yes	0.76 (0.36–1.56)	0.79 (0.34–1.82)	0.56 (0.30–1.05)	0.41^*∗*^ (0.20–0.86)
Having chronic disease				
One	*Reference*			
Two	1.03 (0.55–1.85)	1.03 (0.54–1.97)	1.00 (0.61–1.65)	0.98 (0.57–1.66)
Three	0.54 (0.19–1.49)	0.55 (0.18–1.62)	0.77 (0.35–1.66)	0.83 (0.37–1.88)
More than three	0.00	0.00	0.97 (0.35–2.66)	1.07 (0.37–3.05)
Smoking status				
Nonsmoker	*Reference*			
Ever smoker	1.53 (0.94–2.50)	1.39 (0.82–2.37)	1.69 (0.87–3.29)	1.79 (0.86–3.70)
Current smoker	0.93 (0.57–1.52)	0.90 (0.53–1.54)	1.79 (0.86–3.70)	2.22^*∗*^ (1.13–4.35)
Quitting smoking				
1–10 years	*Reference*			
11–20 years	1.27 (0.48–3.32)	1.19 (0.36–3.90)	1.80 (0.38–8.53)	1.15 (0.12–19.07)
21–30 years	3.20 (0.98–10.41)	2.23 (0.46–10.83)	0.45 (0.04–4.50)	0.00
>30 years	0.60 (0.06–5.70)	1.17 (0.08–17.02)	0.00	0.00
Drinking status				
Nondrinker	*Reference*			
Ever drinker	1.70^*∗*^ (1.01–2.86)	1.61 (0.92–2.81)	1.07 (0.50–2.27)	0.97 (0.44–2.14)
Current drinker	2.48^*∗*^ (1.54–3.99)	2.16^*∗*^ (1.28–3.64)	2.51^*∗*^ (1.21–5.24)	2.73^*∗*^ (1.28–5.83)
Quitting drinking				
1–10 years	*Reference*			
11–20 years	1.22 (3.74–3.97)	1.59 (0.39–6.53)	1.33 (2.60–6.82)	1.58 (0.16–14.73)
21–30 years	1.34 (0.30–5.91)	1.44 (0.26–7.83)	0.80 (0.07–8.47)	0.65 (0.03–10.78)
>30 years	0.00	0.00	0.00	0.00

Adjusted OR: province, ages, marital status, income, and education level.

^*∗*^ *p* < .05.

**Table 3:** Binary logistic regression for various categories of quality of life.

	QoL											
	SAB		AUT		PPF		SOP		DAD		INT	
	Crude ORs	Adjusted ORs	Crude ORs	Adjusted ORs	Crude ORs	Adjusted ORs	Crude ORs	Adjusted ORs	Crude ORs	Adjusted ORs	Crude ORs	Adjusted ORs
Male												
Drinking Status												
Nondrinker	*Reference*											
Ever drinker	1.32 (0.73–2.39)	1.14 (0.61–2.15)	1.36 (0.81–2.28)	1.34 (0.77–2.31)	0.78 (0.44–1.37)	0.60 (0.32–1.12)	1.61 (0.97–2.67)	1.31 (0.76–2.27)	1.24 (0.70–2.20)	1.27 (0.70–2.30)	1.10 (0.63–1.91)	1.05 (0.58–1.88)
Current drinker	2.17^*∗*^ (1.30–3.63)	1.70 (0.97–3.00)	1.60 (0.99–2.59)	1.49 (0.88–2.52)	1.03 (0.62–1.70)	0.76 (0.43–1.34)	1.57 (0.97–2.53)	1.13 (0.66–1.92)	1.91^*∗*^ (1.15–3.16)	1.85^*∗*^ (1.07–3.17)	1.39 (0.84–2.29)	1.25 (0.72–2.16)
Female												
Nondrinker	*Reference*											
Ever drinker	1.64 (0.83–3.27)	1.55 (0.75–3.19)	0.70 (0.29–1.70)	0.70 (0.28–1.73)	1.06 (0.51–2.19)	0.76 (0.35–1.64)	1.21 (0.60–2.44)	0.79 (0.37–1.71)	1.81 (0.94–3.48)	1.68 (0.85–3.30)	0.08^*∗*^ (0.01–0.58)	0.08^*∗*^ (0.01–0.64)
Current drinker	2.66^*∗*^ (1.27–5.56)	2.94^*∗*^ (1.37–6.28)	1.04 (0.42–2.58)	1.01 (0.40–2.54)	0.68 (0.25–1.79)	0.59 (0.22–1.63)	0.85 (0.34–2.11)	0.74 (0.28–1.94)	3.44^*∗*^ (1.69–7.01)	3.78^*∗*^ (1.82–7.88)	1.35 (0.61–2.96)	1.34 (0.59–3.04)

Adjusted OR: province, ages, marital status, income, and education level.

^*∗*^ *p* < .05.
